# 1-Meth­oxy-2-methyl­propan-2-aminium 2,2,2-trifluoro­acetate

**DOI:** 10.1107/S1600536810019100

**Published:** 2010-05-29

**Authors:** Kai Wang, Yu-Feng Li, Xiang-Hua Song, Mei-Li Feng, Hong-Jun Zhu

**Affiliations:** a Department of Applied Chemistry, College of Science, Nanjing University of Technology, Nanjing 210009, People’s Republic of China

## Abstract

In the title salt, C_5_H_14_NO^+^·C_2_F_3_O_2_^−^, the cation and anion are linked by N—H⋯O and O—H⋯N hydrogen bonds, generating a three-dimensional network.

## Related literature

The title compound is an inter­mediate in the synthesis of 1-meth­oxy-*N*,2-dimethyl­propan-2-amine. For the synthesis of the title compound, see: Maeda *et al.* (2004 ▶). For bond-length data, see: Allen *et al.* (1987 ▶).
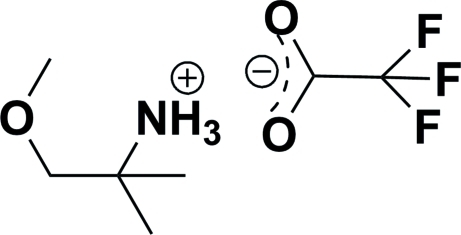


## Experimental

### 

#### Crystal data

C_5_H_14_NO^+^·C_2_F_3_O_2_^−^
*M* *_r_* = 217.19
Orthorhombic, 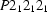

*a* = 6.6680 (13) Å
*b* = 8.9900 (18) Å
*c* = 17.862 (4) Å
*V* = 1070.7 (4) Å^3^
*Z* = 4
Mo *K*α radiation
μ = 0.14 mm^−1^
*T* = 293 K
0.30 × 0.10 × 0.10 mm

#### Data collection

Enraf–Nonius CAD-4 diffractometer
Absorption correction: ψ scan (North *et al.*, 1968 ▶) *T*_min_ = 0.961, *T*_max_ = 0.987
1861 measured reflections
1150 independent reflections
784 reflections with *I* > 2σ(*I*)
*R*_int_ = 0.038
3 standard reflections every 200 reflections  intensity decay: 1%

#### Refinement

*R*[*F* ^2^ > 2σ(*F* ^2^)] = 0.060
*wR*(*F* ^2^) = 0.183
*S* = 1.01
1150 reflections
121 parameters
2 restraints
H-atom parameters constrained
Δρ_max_ = 0.39 e Å^−3^
Δρ_min_ = −0.27 e Å^−3^

### 

Data collection: *CAD-4 Software* (Enraf–Nonius, 1985 ▶); cell refinement: *CAD-4 Software*; data reduction: *XCAD4* (Harms & Wocadlo, 1995 ▶); program(s) used to solve structure: *SHELXS97* (Sheldrick, 2008 ▶); program(s) used to refine structure: *SHELXL97* (Sheldrick, 2008 ▶); molecular graphics: *SHELXTL* (Sheldrick, 2008 ▶); software used to prepare material for publication: *SHELXTL*.

## Supplementary Material

Crystal structure: contains datablocks I, global. DOI: 10.1107/S1600536810019100/jh2152sup1.cif

Structure factors: contains datablocks I. DOI: 10.1107/S1600536810019100/jh2152Isup2.hkl

Additional supplementary materials:  crystallographic information; 3D view; checkCIF report

## Figures and Tables

**Table 1:** Hydrogen-bond geometry (Å, °)

*D*—H⋯*A*	*D*—H	H⋯*A*	*D*⋯*A*	*D*—H⋯*A*
N—H0*A*⋯O2	0.89	1.99	2.854 (5)	163
N—H0*B*⋯O2^i^	0.89	2.00	2.859 (5)	161
N—H0*C*⋯O3^ii^	0.89	1.92	2.802 (6)	169

Symmetry codes: (i) 
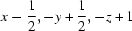
; (ii) 
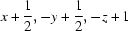
.

**Fig. 1.**
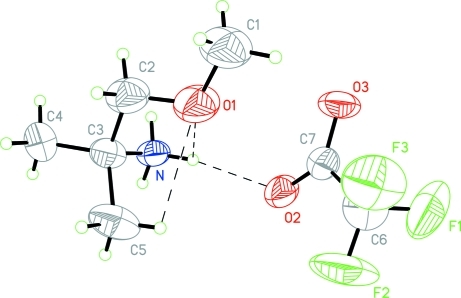
The molecular structure of (I), with the atom-numbering scheme. Displacement ellipsoids are drawn at the 50% probability level.

**Fig. 2.**
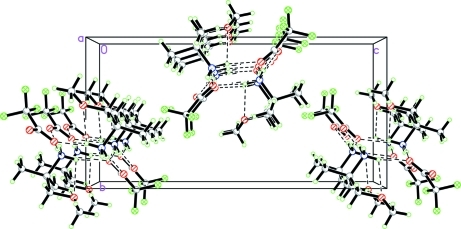
A packing diagram of (I). Hydrogen bonds are shown as dashed lines.

## supplementary crystallographic information

### Comment

The title compound, 1-methoxy-2-methylpropan-2-aminium 2,2,2-trifluoroacetate is an important intermediate for the synthesis of 1-methoxy-*N*,2-dimethylpropan-2-amine. We herein report its crystal structure.

The molecular structure of (I) is shown in Fig. 1, and the selected geometric parameters are given in Table 1. The bond lengths and angles (Table 1) are within normal ranges (Allen *et al.*, 1987).

In the molecule of (I), (Fig.1), the bond lengths (Allen *et al.*, 1987) and angles are within normal ranges. The crystal of this compound was connected together *via* C—H···O, and N—H···O inter- and intramolecular hydrogen bonds to form a three dimensional network, which seems to be very effective in the stabilization of the crystal structure.

As can be seen from the packing diagram, (Fig. 2), the molecules are stacked along the *a* axis.

### Experimental

The title compound, (I) was synthesized according to the literature (Maeda *et al.*, 2004). The crystals were obtained by dissolving (I) (0.52 g, 2.4 mmol) in 25 ml me thanol and evaporating the solvent slowly at room temperature for about 4 d.

### Refinement

H atoms bonded to N and O atoms were located in a difference map and refined with distance restraints of O—H = 0.85 (2) and N—H = 0.90 (2) Å, and with *U*_iso_(H) = 1.2*U*_eq_(N,*O*). Other H atoms were positioned geometrically and refined using a riding model, with C—H = 0.96–0.97 (2) Å, *U*_iso_(H) = *1.5U*_eq_(C). Friedel pairs were merged.

### Crystal data

**Table:**

C_5_H_14_NO^+^·C_2_F_3_O_2_^−^	*F*(000) = 456
*M**_r_* = 217.19	*D*_x_ = 1.347 Mg m^−^^3^
Orthorhombic, *P*2_1_2_1_2_1_	Mo *K*α radiation, λ = 0.71073 Å
Hall symbol: P 2ac 2ab	Cell parameters from 25 reflections
*a* = 6.6680 (13) Å	θ = 9–12°
*b* = 8.9900 (18) Å	µ = 0.14 mm^−^^1^
*c* = 17.862 (4) Å	*T* = 293 K
*V* = 1070.7 (4) Å^3^	Block, colourless
*Z* = 4	0.30 × 0.10 × 0.10 mm

### Data collection

**Table:**

Enraf–Nonius CAD-4 diffractometer	784 reflections with *I* > 2σ(*I*)
Radiation source: fine-focus sealed tube	*R*_int_ = 0.038
graphite	θ_max_ = 25.3°, θ_min_ = 2.3°
ω/2θ scans	*h* = −7→8
Absorption correction: ψ scan (North *et al.*, 1968)	*k* = 0→10
*T*_min_ = 0.961, *T*_max_ = 0.987	*l* = 0→21
1861 measured reflections	3 standard reflections every 200 reflections
1150 independent reflections	intensity decay: 1%

### Refinement

**Table:**

Refinement on *F*^2^	Primary atom site location: structure-invariant direct methods
Least-squares matrix: full	Secondary atom site location: difference Fourier map
*R*[*F*^2^ > 2σ(*F*^2^)] = 0.060	Hydrogen site location: inferred from neighbouring sites
*wR*(*F*^2^) = 0.183	H-atom parameters constrained
*S* = 1.01	*w* = 1/[σ^2^(*F*_o_^2^) + (0.1*P*)^2^ + 0.350*P*] where *P* = (*F*_o_^2^ + 2*F*_c_^2^)/3
1150 reflections	(Δ/σ)_max_ < 0.001
121 parameters	Δρ_max_ = 0.39 e Å^−^^3^
2 restraints	Δρ_min_ = −0.27 e Å^−^^3^

### Special details

**Table:**

Geometry. All e.s.d.'s (except the e.s.d. in the dihedral angle between two l.s. planes) are estimated using the full covariance matrix. The cell e.s.d.'s are taken into account individually in the estimation of e.s.d.'s in distances, angles and torsion angles; correlations between e.s.d.'s in cell parameters are only used when they are defined by crystal symmetry. An approximate (isotropic) treatment of cell e.s.d.'s is used for estimating e.s.d.'s involving l.s. planes.
Refinement. Refinement of *F*^2^ against ALL reflections. The weighted *R*-factor *wR* and goodness of fit *S* are based on *F*^2^, conventional *R*-factors *R* are based on *F*, with *F* set to zero for negative *F*^2^. The threshold expression of *F*^2^ > σ(*F*^2^) is used only for calculating *R*-factors(gt) *etc*. and is not relevant to the choice of reflections for refinement. *R*-factors based on *F*^2^ are statistically about twice as large as those based on *F*, and *R*- factors based on ALL data will be even larger.

### Fractional atomic coordinates and isotropic or equivalent isotropic displacement parameters (Å^2^)

**Table:**

	*x*	*y*	*z*	*U*_iso_*/*U*_eq_	
N	0.4436 (6)	0.2188 (5)	0.4225 (2)	0.0507 (10)	
H0A	0.4554	0.1919	0.4702	0.076*	
H0B	0.3281	0.2667	0.4159	0.076*	
H0C	0.5450	0.2784	0.4102	0.076*	
O1	0.2833 (6)	−0.0471 (5)	0.4723 (2)	0.0789 (13)	
C1	0.1216 (11)	−0.1286 (9)	0.5000 (4)	0.093 (2)	
H1A	0.1419	−0.1486	0.5522	0.139*	
H1B	0.1112	−0.2209	0.4732	0.139*	
H1C	0.0003	−0.0725	0.4935	0.139*	
C2	0.2708 (9)	−0.0130 (8)	0.3959 (3)	0.0674 (16)	
H2A	0.1465	0.0392	0.3857	0.081*	
H2B	0.2719	−0.1040	0.3667	0.081*	
C3	0.4477 (8)	0.0837 (6)	0.3739 (3)	0.0557 (12)	
C4	0.4273 (10)	0.1332 (8)	0.2930 (3)	0.0755 (18)	
H4A	0.2998	0.1808	0.2861	0.113*	
H4B	0.4365	0.0482	0.2607	0.113*	
H4C	0.5328	0.2020	0.2812	0.113*	
C5	0.6472 (9)	0.0065 (9)	0.3891 (4)	0.084 (2)	
H5A	0.6533	−0.0230	0.4407	0.126*	
H5B	0.7554	0.0737	0.3784	0.126*	
H5C	0.6586	−0.0799	0.3578	0.126*	
F1	0.5540 (9)	0.0216 (7)	0.7374 (2)	0.139 (2)	
F2	0.7402 (6)	−0.0271 (7)	0.6453 (4)	0.159 (3)	
F3	0.4744 (7)	−0.1451 (5)	0.6637 (3)	0.1188 (17)	
O2	0.5367 (5)	0.1809 (4)	0.5773 (2)	0.0642 (10)	
O3	0.2549 (5)	0.1057 (5)	0.6350 (2)	0.0685 (11)	
C6	0.5543 (12)	−0.0119 (9)	0.6655 (4)	0.088	
C7	0.4376 (8)	0.1040 (6)	0.6208 (3)	0.0526 (12)	

### Atomic displacement parameters (Å^2^)

**Table:**

	*U*^11^	*U*^22^	*U*^33^	*U*^12^	*U*^13^	*U*^23^
N	0.0321 (18)	0.067 (2)	0.053 (2)	−0.004 (2)	−0.0039 (18)	0.008 (2)
O1	0.058 (2)	0.095 (3)	0.084 (3)	−0.028 (2)	−0.0068 (19)	0.018 (2)
C1	0.068 (4)	0.104 (5)	0.106 (4)	−0.029 (4)	0.003 (4)	0.024 (5)
C2	0.053 (3)	0.069 (4)	0.080 (4)	−0.011 (3)	−0.006 (3)	−0.009 (3)
C3	0.039 (2)	0.067 (3)	0.062 (3)	0.001 (3)	0.004 (2)	−0.004 (3)
C4	0.069 (4)	0.105 (4)	0.053 (3)	0.001 (4)	0.008 (3)	−0.011 (3)
C5	0.052 (3)	0.086 (5)	0.114 (5)	0.018 (4)	0.017 (3)	0.004 (4)
F1	0.149 (4)	0.191 (5)	0.078 (3)	0.032 (5)	−0.040 (3)	0.025 (3)
F2	0.048 (2)	0.199 (6)	0.230 (6)	0.051 (3)	0.044 (3)	0.126 (5)
F3	0.112 (4)	0.077 (2)	0.167 (4)	0.017 (3)	0.007 (3)	0.035 (3)
O2	0.0486 (19)	0.088 (3)	0.056 (2)	−0.018 (2)	−0.0095 (17)	0.021 (2)
O3	0.0305 (18)	0.090 (3)	0.085 (3)	0.0088 (19)	0.0031 (16)	0.021 (2)
C6	0.088	0.088	0.088	0.000	0.000	0.000
C7	0.049 (3)	0.060 (3)	0.049 (3)	0.002 (3)	−0.003 (2)	0.010 (3)

### Geometric parameters (Å, °)

**Table:**

N—C3	1.493 (7)	C3—C5	1.525 (8)
N—H0A	0.8900	C4—H4A	0.9600
N—H0B	0.8900	C4—H4B	0.9600
N—H0C	0.8900	C4—H4C	0.9600
O1—C1	1.394 (7)	C5—H5A	0.9600
O1—C2	1.402 (7)	C5—H5B	0.9600
C1—H1A	0.9600	C5—H5C	0.9600
C1—H1B	0.9600	F1—C6	1.319 (8)
C1—H1C	0.9600	F2—C6	1.298 (9)
C2—C3	1.517 (8)	F3—C6	1.311 (9)
C2—H2A	0.9700	O2—C7	1.232 (6)
C2—H2B	0.9700	O3—C7	1.244 (6)
C3—C4	1.518 (8)	C6—C7	1.526 (9)
			
C3—N—H0A	109.5	C2—C3—C5	111.8 (4)
C3—N—H0B	109.5	C4—C3—C5	112.4 (5)
H0A—N—H0B	109.5	C3—C4—H4A	109.5
C3—N—H0C	109.5	C3—C4—H4B	109.5
H0A—N—H0C	109.5	H4A—C4—H4B	109.5
H0B—N—H0C	109.5	C3—C4—H4C	109.5
C1—O1—C2	114.4 (5)	H4A—C4—H4C	109.5
O1—C1—H1A	109.5	H4B—C4—H4C	109.5
O1—C1—H1B	109.5	C3—C5—H5A	109.5
H1A—C1—H1B	109.5	C3—C5—H5B	109.5
O1—C1—H1C	109.5	H5A—C5—H5B	109.5
H1A—C1—H1C	109.5	C3—C5—H5C	109.5
H1B—C1—H1C	109.5	H5A—C5—H5C	109.5
O1—C2—C3	109.3 (4)	H5B—C5—H5C	109.5
O1—C2—H2A	109.8	F2—C6—F3	106.6 (7)
C3—C2—H2A	109.8	F2—C6—F1	107.2 (7)
O1—C2—H2B	109.8	F3—C6—F1	103.4 (7)
C3—C2—H2B	109.8	F2—C6—C7	114.4 (6)
H2A—C2—H2B	108.3	F3—C6—C7	113.8 (6)
N—C3—C2	107.6 (4)	F1—C6—C7	110.6 (6)
N—C3—C4	108.2 (5)	O2—C7—O3	130.3 (5)
C2—C3—C4	110.2 (5)	O2—C7—C6	116.1 (5)
N—C3—C5	106.4 (5)	O3—C7—C6	113.6 (5)
			
C1—O1—C2—C3	176.4 (5)	F3—C6—C7—O2	−130.9 (6)
O1—C2—C3—N	−57.2 (6)	F1—C6—C7—O2	113.3 (7)
O1—C2—C3—C4	−175.0 (5)	F2—C6—C7—O3	172.5 (7)
O1—C2—C3—C5	59.3 (6)	F3—C6—C7—O3	49.6 (8)
F2—C6—C7—O2	−8.0 (9)	F1—C6—C7—O3	−66.3 (8)

### Hydrogen-bond geometry (Å, °)

**Table:**

*D*—H···*A*	*D*—H	H···*A*	*D*···*A*	*D*—H···*A*
N—H0A···O1	0.89	2.44	2.766 (6)	102
N—H0A···O2	0.89	1.99	2.854 (5)	163
N—H0B···O2^i^	0.89	2.00	2.859 (5)	161
N—H0C···O3^ii^	0.89	1.92	2.802 (6)	169
C5—H5A···O1	0.96	2.54	2.886 (8)	101

Symmetry codes: (i) *x*−1/2, −*y*+1/2, −*z*+1; (ii) *x*+1/2, −*y*+1/2, −*z*+1.
